# SARS-CoV-2 vaccine breakthrough infection and the evaluation of safety precaution practice before and after vaccination among healthcare workers in South West, Nigeria

**DOI:** 10.1186/s12889-024-18663-y

**Published:** 2024-05-08

**Authors:** Oluwatosin Idowu Oni, Patrick Olanrewaju Osho, Tayelolu Mary Odesanmi, Habeebat Motunrayo Raji, Faith Titilayo Oluranti, Demian Ibina

**Affiliations:** 1 Ondo State Primary Health Care Development Agency, Akure, Ondo State Nigeria; 2 Department of Haematology and Immunology, University of Medical Sciences, Ondo, Ondo State Nigeria; 3 Public Health, University of Medical Sciences, Ondo, Ondo State Nigeria; 4 Department of Statistics, Federal University of Technology, Akure, Nigeria; 5 Suddan United Mission Hospital, Abakaliki, Ebonyi State Nigeria

**Keywords:** COVID-19, Breakthrough infections, Pre and post-vaccination, Nasopharyngeal

## Abstract

**Introduction:**

Worldwide, it has been reported that fully vaccinated people still die of COVID-19-associated symptoms, generating public uncertainty about the safety and effectiveness of the vaccines. Hence, this research is aimed at assessing the incidence of COVID-19 breakthrough infection among vaccinated Health Workers and the possible effect of changes in the practice of post-vaccination safety precautions.

**Method:**

This was a Health facility-based descriptive cross-sectional study. Data were collected using self-administered questionnaires distributed at the participant’s work unit across the selected health facilities. The nasopharyngeal specimen was also obtained from the participants and analysed using STANDARD Q COVID-19 Ag Test rapid chromatographic immunoassay for the detection of antigens to SARS-CoV-2. All data were input and analyzed using SPSS version 20.

**Results:**

There was a statistically significant relationship between the vaccination status of respondents and the post-vaccination test result (χ^2^ = 6.816, df = 1, *p* = 0.009). The incidence of COVID-19 infection among the vaccinated and unvaccinated HCWs was 2% and 8% respectively. 5 of the 15 respondents who tested positive for COVID-19 had been fully vaccinated. However, all 5 of them did not practice safety measures after vaccination. None of the respondents who practised safety measures after vaccination tested positive for COVID-19. The remaining 10 respondents that tested positive for COVID-19 had not been vaccinated though they practised safety precautions.

**Conclusion:**

Vaccination and the practice of safety precautions will go a long way to preventing future COVID-19 breakthrough infections.

**Supplementary Information:**

The online version contains supplementary material available at 10.1186/s12889-024-18663-y.

## Introduction

The novel Coronavirus (COVID-19), caused by severe acute respiratory syndrome virus 2 (SARS-CoV-2) [1] was declared a public health emergency of international concern by the World Health Organization on January 30, 2020 [2].

The Federal Ministry of Health reported the first case of COVID-19 in Nigeria on February 27, 2020, and by March 30, 2020, the Nigerian Government implemented the lockdown strategy as an essential measure to reduce the spread of the virus across the country [3].

As part of the government’s all-round response to the COVID-19 epidemic, the President of the Federal Republic of Nigeria equally announced the compulsory use of face masks by anyone going out in public [4].

As of June 2022, over five hundred thirty-one million confirmed cases of COVID-19 had been reported worldwide, with over six million related deaths and over eleven billion vaccine doses administered [5]. In Nigeria, over two hundred fifty-six thousand cases have been reported with more than three thousand deaths [6].

The instant strategy adopted by most countries around the world to reduce the transmissibility of the disease was Non-Pharmaceutical Interventions (NPIs), like enforcing masks policy, hands sanitization, social distancing, travel restrictions, schools’ closure, and partial or complete lockdowns [7]. In the year 2020, the Nigerian Government as well put up these NPIs to control the spread of the virus. However, enforcing these measures was met with several challenges among the general population [8]. NPIs were able to slow down the progression of the disease, but the most potent strategy to restrict the pandemic and reduce the mortality and morbidity rates is the development of safe, effective and readily available vaccines.

With a case fatality rate of about 1.2% globally [5] and also in Nigeria [6], it appeared vaccination would be the utmost effective means of reducing the burden of the COVID-19 disease.

The World Health Organization (WHO) emphasizes the prioritization of policies promoting voluntary vaccination, such as public information campaigns, over mandatory vaccination policies [9]. Nevertheless, COVID-19 vaccine mandates are regarded as a reasonable measure when alternative approaches prove insufficient in achieving satisfactory vaccination coverage. Both strategies may be implemented concurrently if found beneficial. Healthcare workers (HCWs) are frequently the focus of vaccine mandates, partly due to their moral responsibility to prevent harm to patients and other considerations related to their roles [10].

In a study conducted by Biswas et al. [11], global COVID-19 vaccination hesitancy among healthcare workers varied between 4.3% and 72%, with an average of 22.51% across all studies involving 76,471 participants. The primary reasons for hesitancy, as indicated included concerns about vaccine safety, efficacy, and potential side effects. Factors such as a heightened perceived risk of contracting COVID-19, direct patient care responsibilities, and a history of influenza vaccination were also identified as contributors to an increased likelihood of COVID-19 vaccination acceptance.

To address the pandemic, the Nigerian government mandated that all federal government employees, including healthcare workers, must receive the Covid-19 vaccine [12].

Publishing the genetic sequence of SARS-CoV-2 on 11 January 2020, generated an urgent international response to hasten the development of a preventive COVID-19 vaccine [13].

The rapidly growing incidence of COVID-19 in the year 2020 inspired international coalitions and government efforts to urgently consolidate resources towards the production of multiple vaccines within the shortest possible time [14]. By December 2021, several vaccines had been given emergency use listing by the WHO [15] out of which the following have been used in Nigeria for the prevention of COVID-19.

- Pfizer (mRNA) vaccine.
- Moderna (mRNA-1273) vaccines.
- AstraZeneca vaccines.
- Janssen vaccine.

The mRNA-based vaccines do not integrate with the host cell genome and can produce pure viral protein. The technology involved in the development of this vaccine avoids the time-consuming normalization processes, this enhances rapid commercial production, which is particularly important during a pandemic [16].

### The Oxford/AstraZeneca Covid-19 vaccine

A viral vector vaccine, which uses a safe virus that cannot cause disease but serves as a platform to produce coronavirus proteins to generate an immune response.

### The Janssen Ad26.COV2.S Covid-19 vaccine

The Johnson and Johnson vaccine uses a disabled adenovirus to deliver instructions in the host, unlike Pfizer which uses mRNA.

The first shipment of COVID-19 vaccines arrived at the Nnamdi Azikiwe International Airport Nigeria on 2 March 2021 (about 4,000,000 Oxford-AstraZeneca vaccines doses) [17] and vaccinations began three days later on 5 March, 202 [18]. As of 16th June 2022, 11.9 billion vaccine doses had been given globally with 5.19 billion partially vaccinated and 4.73 billion people fully vaccinated [5].

In Nigeria, as of 16th June 2022, about 28 million people had been partially vaccinated representing a 25.4% proportion while about 20 million people had been fully vaccinated representing a vaccination proportion of 18.8% [19].

To ease up COVID-19 restrictions across the country, the Nigerian Government on 31st March 2022 declared that the use of face masks was no longer compulsory (https://tribuneonlineng.com/covid-19-wearing-of-face-masks-in-public-places-now-optional-says-fg/).

Regardless of the progress made by the development of COVID-19 vaccines, there are still important issues that need to be addressed. Other than misinformation, lack of information, and anti-vaccine opinions, there are several, carefully designed conspiracy theories around the Covid-19 virus. Across the world, it has been reported that people who were fully vaccinated still died of COVID-19-associated symptoms, which has also deepened public uncertainty about the safety and effectiveness of the vaccines [20]. For this study, breakthrough infection refers to a SARS-CoV-2 infection that occurs after completion of the recommended COVID-19 vaccine series. Hence, this research is aimed at assessing the incidence of COVID-19 infection among vaccinated health workers and the possible changes in the attitude of these health workers to COVID-19 safety precautions before and after vaccination. This will help us understand the protective potency of the various vaccines against COVID-19 infection which will in turn influence the acceptance of vaccines among the general public. It is also anticipated that evidence on breakthrough risk can inform public health policies, including recommendations of additional primary doses and the need to always practice universal safety precautions.

## Method

### Study area

The study was carried out in Ondo State, South West Nigeria. Ondo state was created in 1976 out of the old Ondo province.

As stated by the Department of Planning, Research and Statistics, Ondo State Ministry of Health, Akure, there are 589 Primary Health Care Centres, 5 Secondary Hospitals and 1 Teaching Hospital in Ondo State [21].

This was a health facility-based descriptive cross-sectional study aimed at understanding the attitude of health workers to the practice of safety precautions after vaccination and the occurrence of covid-19 breakthrough infections.

For this study, the only Teaching Hospital in the State as well as three (3) General hospitals – one (1) General Hospital in each of the three regions (central, southern and northern senatorial regions) of the state and ninety-seven (97) Primary Health Centres (PHCs)were selected by simple random sampling (balloting).

### Study participants

#### Inclusion criteria

Health workers that had received minimum of 2 doses (AstraZeneca or Moderna) or 1 dose of Johnson and Johnson and took the last dose within the last 6 months from the selected Primary Health Care Centres, Secondary Hospitals and Teaching Hospital were included in the study.

Health Workers who had not received any dose of vaccination were also included in the study for comparison.

The index date was defined as the date of receipt of the first vaccine dose while fully vaccinated was defined as ≥ 14 days after receipt of the second vaccine dose. The cadres of health workers captured were: Doctors, Pharmacists, Nurses/Midwives, Laboratory Scientists/Technicians, Community Health Officers (CHO) and Community Health Extension Workers (CHEW).

#### Exclusion criteria

All non-health workers like administrative officers were excluded from the study.

### Sample size and sampling procedure

The sample size was calculated using the Leslie Fischer formula for sample size determination in health studies [21].

$$n=Z\Lambda2pq/d^2$$

P is the prevalence, q = 1-p, Z = Standard normal deviation which corresponds to the 95% confidence level (1.96), d = degree of accuracy desired (0.05).

The prevalence (p) of COVID-19 vaccination in Ondo State, confidence level and marginal error were given to be 55.5% [8], 95% and 5% respectively. The final sample size was 344 after adjustment for a 12% non-response rate.

### Data collection

In August 2022, a structured questionnaire designed and used to obtain data from the participants. A pilot test was carried out to ascertain the validity of the questions. Necessary reviews were made to the questions after the pilot test. An informed consent form was signed by each participant before administering the questionnaires. Subsequent explanations and permission to carry out the COVID-19 test were obtained from the participants with a unique identification number given to individuals to ensure confidentiality.

### Collection of nasopharyngeal samples and running of COVID-19 test

A sterile swab was inserted into the nostril of the participants and swabbed over the surface of the posterior nasopharynx to obtain a nasopharyngeal specimen. STANDARD Q COVID-19 Ag Test rapid chromatographic immunoassay for the detection of antigens to SARS-CoV-2 present in the human nasopharynx was used to test for the COVID-19 Status of the participants.

### Data management

Information from the structured questionnaire and the COVID-19 result details were entered into a Statistical Package for the Social Sciences (SPSS) version 21 program. Data cleaning was done to ensure that all variables were correctly entered before the analysis. Descriptive analysis was carried out for all categorical variable while the test of means was carried out to establish the existence of significant or no significant relationship between variables (a *P* value < 0.05 was considered statistically significant). Binary logistic regression was performed to identify socio-demographic factors independently associated with the dependent variable. The strength of association was measured using odds ratio, and 95% confidence intervals.

### Ethical approval

Ethical approval was obtained from the Research Ethical Committee, University of Medical Sciences, Ondo, Ondo State, Nigeria. (NHREC/TR/UNIMED-HREC-Ondo St/22/06/21).

Also, each participant was requested to carefully go through the informed consent form and sign it before proceeding to answer the questions. All procedures were carried out following relevant guidelines and regulations on research involving human subjects according to the Helsinki Declaration [22].

## Results

Three hundred fifty questionnaires were distributed out of which 341 respondents answered the questions and agreed to carry out COVID-19 investigation. This represented a 97.43% responsive rate.

Table 1 shows the background summary statistics of 341 HCWs who responded to the survey. The most reflected age range of the respondents was 21–25 years. The majority of survey respondents were females (72.1%) and Nursing cadre (71%).

**Table 1:** Socio-demographic characteristics of respondents (*N* = 341)

Variables		N (%)
**Age of respondents**	< 20 years	55(16.1)
	21-25 years	103(30.2)
	26-30 years	55(16.1)
	31-35 years	54(15.8)
	36-40 years	38(11.1)
	> 40 years	36(10.6)
**Sex**	Male	121(35.5)
	Female	220(64.5)
**Years of experience of Health Workers**	1–3 years	172(50.4)
	4–6 years	65(19.1)
	7–9 years	51(15.0)
	> 10 years	53(15.5)
**Cadre of respondents**	Doctor	44(12.9)
	Pharmacist	53(15.5)
	Nurse/midwife	111(32.6)
	Lab scientist/technician	69(20.2)
	CHO	20(5.9)
	CHEW	44(12.9)

From Table 2, all the respondents practiced safety measures before vaccination while just over 50% reported to have practised safety measures after vaccination.

**Table 2:** COVID-19 vaccination knowledge, vaccination status, practice of safety precautions (pre and post vaccination) and covid-19 status

Question	Options	Frequency (n)	Percent (%)
Have you ever heard about COVID-19?	Yes	341	100.0
	No	0	0.0
Mention some preventive measures you know	Physical distance	82	24.0
	Nose mask use	97	28.4
	Vaccination	92	27.0
	Use of Hand sanitizer/regular washing of hands	70	20.5
	Others (Specify):	0	0.0
Which of these safety measures did you practice before vaccination?	Physical distance	176	51.6
	Nose mask use	98	28.7
	Use of Hand sanitizer/regular washing of hands	67	19.6
	Others (Specify):	0	0.0
Are you aware of COVID-19 vaccines	Yes	341	100
	No	0	0.0
How potent do you think the various COVID-19 vaccines are in preventing subsequent infection	Highly potent	97	28.4
	Moderately potent	78	22.9
	Not potent	96	28.2
	I don’t Know	70	20.5
Have you taken COVID-19 vaccine?	Yes	221	64.8
	No	120	35.2
How many doses	0 dose (Not vaccinated)	120	35.2
	1 dose (partially vaccinated)	0	0.0
	2 doses (fully vaccinated)	179	52.5
	Booster dose	42	12.3
When did you receive your last dose	< 1 Month	58	26.2
	1–3 Months	62	28.1
	4–6 Months	101	45.7
	> 6 Months	0	0.0
Which vaccine did you receive?	Astrazeneca + Astrazeneca	65	29.4
	Astrazeneca + Pfizer	39	17.6
	Moderna + Moderna	25	11.3
	Moderna + Pfizer	20	9.0
	Pfizer & Pfizer	17	7.7
	Johnson And Johnson (1 Dose)	13	5.9
	Astrazeneca + Astrazeneca + Pfizer	18	8.1
	Moderna + Moderna + Pfizer	7	3.2
	Johnson And Johnson + Pfizer	5	2.3
	Astrazeneca + Pfizer + Pfizer	12	5.4
Did you practice safety measures after vaccination?	Yes	261	76.5
	No	80	23.5
	Don’t know	0	0.0
Which measures?	Physical distancing	66	19.4
	Nose mask	31	9.1
	Use of hand sanitizer or regular washing of hands	164	48.1
	Not Applicable	80	23.5
Do you agree with us carrying out your COVID-19 test?	Yes	341	100.0
	No	0	0.0
Nasopharyngeal sample collected	Yes	341	100.0
	No	0	0.0
COVID-19 result	Positive	15	4.4
	Negative	326	95.6

From Table 3, there was a statistically significant relationship between the cadre of health workers and the practice of post-vaccination safety measures (*P* < 0.005) with the laboratory personnel and the Community Health Officers having the highest and lowest practice of post-vaccination safety measures respectively. Other factors like age, years of working experience and gender of the respondent did not have any significant association with the practice of post-vaccination safety measures.

**Table 3:** Association between respondents’ socio-demographic characteristics and practice of post-vaccination safety precautions

Variables	Did you practice safety measures after vaccination?		χ^2^	df	*P*-value
	**Yes**	**No**			
^*^Age (year)					
< 20	39(60.0)	18(40.0)	5.308	5	0.379
21–25	83(77.1)	20(22.9)			
26–30	44(62.85)	11(37.15)			
31–35	37(50.0)	17(50.0)			
36–40	28(44.4)	10(55.6)			
> 40	30(62.5)	6(37.5)			
^*^Gender					
Male	95(59.0)	26(41.0)	0.407	1	0.524
Female	166(66.3)	54(33.7)			
^*^Cadre of respondents					
Doctor	28(27.3)	16(72.7)	13.397	5	^*^0.020
Pharmacist	37(40.7)	16(59.3)			
Nurse/midwife	94(77.5)	17(22.5)			
Lab Scientist/Technician	56(80.6)	13(19.4)			
CHO	12(20.0)	8(80.0)			
CHEW	34(58.3)	10(41.7)			
^*^Years of working experience					
1–3 years	127(69.7)	45(30.0)	1.557	3	0.669
4–6 years	51(60.0)	14(40.0)			
7–9 years	40(42.9)	11(57.1)			
10 or more year	433(56.5)	10(43.5)			

^*^Statistically significant *p* < 0.05

Table 4 shows no statistically significant relationship between age and years of working experience and the COVID-19 post-vaccination test result. However, there was a significant relationship between the gender as well as cadre of respondents and the COVID-19 test result with the Doctors having the highest percentage incidence (4.5%).

**Table 4:** Association between respondents’ socio-demographic characteristics and COVID-19 post-vaccination test results

Variables	Post-vaccination covid-19 test result		χ^2^	df	*P*-value
	**Positive**	**Negative**			
^*^Age (year)					
< 20	5(8.6)	50(91.4)	10.987	5	0.052
21–25	8(2.4)	95(97.6)			
26–30	1(0.0)	54(100.0)			
31–35	0(0.0)	54(100.0)			
36–40	0(0.0)	38(100.0)			
> 40	1(55.6)	35(44.4)			
^*^Gender					
Male	12(4.9)	109(95.1)	13.582	1	^*^0.000
Female	3(1.3)	217(98.7)			
^*^Cadre of respondents					
Doctor	3(4.5)	41(95.5)	13.934	5	^*^0.016
Pharmacist	7(0.0)	46(100.0)			
Nurse/midwife	2(1.4)	109(98.6)			
Lab Scientist/Technician	2(3.0)	67(97.0)			
CHO	0(0.0)	20(100.0)			
CHEW	1(1.1)	43(98.9)			
^*^Years of working experience					
1–3 years	9(3.5)	163(96.5)	1.062	3	0.786
4–6 years	3(0.0)	62(100.0)			
7–9 years	1(0.0)	50(100.0)			
10 or more year	2(0.0)	51(100.0)			

^*^Statistically significant *p* < 0.05

From Table 6, there was no statistically significant relationship between the vaccine combination received and the post-vaccination test result.

## Discussion

To the best of our knowledge, this study is the first to assess the incidence of Breakthrough Infections (BTIs) and the practice of safety precautions after vaccination in Nigeria.

About 64% of the respondents were female. Also, the majority of the respondents were Nurses or Midwives. This corroborates the gender distribution of the respondents because there are more female gender practicing the Nursing profession compared to the male gender [18]. The majority of the respondents were young employees with over 30% of the respondents falling between the 21 to 25 years age range. This was also reflected in the years of working experience of the respondents. The majority of them had between 1 to 3 years of working experience as health workers.

All the respondents have heard about COVID-19 as well as COVID-19 vaccination and they all had good knowledge about preventive measures. Equally, all the respondents practised safety measures before receiving the COVID-19 vaccine. This was because, before the commencement of vaccination, safety precautions were the only way of preventing an infection. Remarkably, the majority of the Healthcare Workers reported physical distancing as their major means of observing safety measures while about 28% of them were committed to the use of face masks and just 19.6% of the participants practised the use of hand sanitiser or regular washing of hands.

Expectedly, all the respondents were well aware of covid-19 vaccine. However, about 20% of the respondents could not ascertain the efficacy of the vaccines while 28% of them believed that the vaccines were not potent. Though, another 28% opined that the vaccines were highly potent.

Almost 70% of the respondents had been fully vaccinated over 3 months from the index date. This percentage was quite higher than the 55.5% vaccine acceptance rate reported in Ondo State by Olu-Abiodun et al., 2022 [8]. This was due to the difference in the study population (Health care Workers and the general population). Interestingly, 19.0% of the vaccinated health care workers had received booster doses of the vaccine.

By vaccine combination, about 29% of the vaccinated respondents received AstraZeneca + AstraZeneca (complete vaccination). This was not unexpected as AstraZeneca was the first vaccine shipped into Nigeria on 2nd March, 2021 [13].

In contrast to the practice of safety measures before vaccination, 23.5% of the respondents reported not practising safety precautions after vaccination. In like manner, the most practised safety precaution after vaccination was regular hand washing or the use of hand sanitiser which was the least practised before vaccination. This may be due to vaccinated individuals relaxing on the use of nose masks and physical distancing but the idea of regular hand washing goes beyond covid-19 protection. Physical distancing, the most practised safety measure before vaccination was reported to be an effective means of preventing infection though the reported 2 m may not be sufficient to prevent the transmission of respiratory particles. Also, face-covering can mitigate the spread of the COVID-19 but are not capable of providing maximum protection against the infection as reported by Chea et al*.,* 2021 [23].

Fifteen out of the three hundred forty-one participants representing 4.4% tested positive for covid-19 test. From Table 4, there was no significant relationship between the COVID-19 test result and the age of the respondents. Though, the majority of the positive participants were between the 21–25 age ranges. Also, the years of working experience did not have a statistically significant relationship with the COVID-19 test result. However, there was a statistically significant relationship between gender and the covid-19 test result as well as the cadre of respondents and covid -19 test result. The majority of the Positive respondents to the COVID-19 test were male and Doctors representing the gender and cadre respectively.

There was a statistically significant relationship between the vaccination status of respondents and the post-vaccination test result from Table 5 (χ^2^ = 6.816, df = 1, *p* = 0.009). The incidence of COVID-19 infection among the vaccinated and unvaccinated HCWs was 2% and 8% respectively. This was in agreement with the study findings by Lee et al.,* 2022,* which indicated a greater risk of infection among the unvaccinated individuals for SARS‐CoV‐2 infection compared to the vaccinated individuals [24].

**Table 5:** Relationship between COVID-19 vaccination status and post-vaccination test result

		Post vaccination COVID-19 test result		Total	**χ**^**2**^	df	*P*-value
		Positive	Negative				
Have you taken COVID-19 vaccine?	Yes	5	216	221	6.816	1	^*^0.009
	No	10	110	120			
Total		15	326	341			

Interestingly, there was a statistically significant relationship between the vaccination status of participants and the post-vaccination COVID-19 test results

Table 6 showed no statistically significant relationship between the vaccine combinations received by the Healthcare Workers and COVID-19 test results. This was similar to the findings by Ayoubkhani et al*., 2022* that there was no statistically significant difference in post-vaccination long covid-19 trajectories between participants who received an adenovirus vector vaccine (like Johnson and Johnson) and those who received an mRNA vaccine (like Pfizer) [25]. However, the highest incidence (8%) of vaccine breakthrough infection was observed among those that received Johnson and Johnson (1 dose for complete vaccination) while HCWs that received Moderna + Pfizer or Pfizer + Pfizer had 0% incidence of COVID-19 vaccine breakthrough infection. This finding articulates the difference in the efficiency of the various vaccine combinations on the prevention of breakthrough infection among people with different local features and virus variants of COVID-19 infection [26]. This finding also agreed with the report by Stouten and colleagues that adenoviral-vector-based vaccines were associated with a higher risk of breakthrough infections, compared to mRNA-based vaccines [27].

**Table 6:** Relationship between COVID-19 vaccine combinations received and post-vaccination test results

		**Post-vaccination covid-19 test result**					**χ**^**2**^	**df**	***P*-value**
	**Vaccine combination**	**Positive**		**Negative**		**Total**			
		**N**	**%**	**N**	**%**				
**Which vaccines did you receive?**	NA	10	8	110	92	120	9.203	10	0.513
	A	1	2	64	98	65			
	B	1	3	38	97	39			
	C	1	4	24	96	25			
	D	0	0	20	100	20			
	E	0	0	17	100	17			
	F	1	8	12	92	13			
	G	1	6	17	94	18			
	H	0	0	7	100	7			
	I	0	0	5	100	5			
	J	0	0	12	100	12			
**Total**		**15**	**4**	**326**	**96**	**341**			

Keys to vaccine combination:

NA = Not Applicable

A = AstraZeneca + AstraZeneca

B = AstraZeneca + Pfizer

C = Moderna + Moderna

D = Moderna + Pfizer

E = Pfizer + Pfizer

F = Johnson and Johnson

G = AstraZeneca + AstraZeneca + Pfizer (booster dose)

H = Moderna + Moderna + Pfizer (booster dose)

I = Johnson and Johnson + Pfizer (booster dose)

J = AstraZeneca + Pfizer + Pfizer (booster dose)

^*^Statistically significant *p* <0.05

From Table 7, there was a significant relationship (*p* < 0.05) between the time after vaccination completion and the testing for COVID-19 infection. About 7% of those that received their last vaccine dose within 1 month of getting tested were positive for covid-19 while just 1% of those that received the last dose of their vaccine between 4 to 6 months before testing was positive for covid-19.

**Table 7:** Relationship between the duration of the last dose before testing and the covid-19 test result

When did you take the last dose ^*^ post vaccination COVID-19 **test result**							
		Post-vaccination COVID-19 test result		Total	**χ**^**2**^	df	*P*-value
		Positive	Negative				
When did you take the last dose	Not applicable	10	110	120	7.807	3	*0.012
	< 1 month	4	54	58			
	1–3 months	0	62	62			
	4–6 months	1	100	101			
Total		15	326	341			

There was a significant association between the duration of the last dose and the test result

^*^Statistically significant *p* < 0.05

About 8% of non-vaccinated healthcare Workers tested positive for COVID-19 while about 2% of fully vaccinated respondents and respondents who had taken booster doses tested positive for the covid-19 test (Table 8). This showed a statistically significant relationship between the number of doses of COVID-19 vaccine received and the COVID-19 post-vaccination test result. Our study indicated that the incidence of COVID-19 breakthrough infection was higher among people who are fully vaccinated but without booster doses compared to fully vaccinated persons with booster doses. This was consistent with findings in other studies [21].

**Table 8:** Number of COVID-19 vaccination doses and the test result of respondents

		Post-vaccination COVID-19 test result		Total	**χ**^**2**^	df	*P*-value
		Positive	Negative				
How many doses of COVID-19 vaccine	0 dose (not vaccinated)	10	110	120			
	2 doses (full vaccination)	4	175	179	6.818	2	^*^0.033
	Booster dose	1	41	42			
Total		15	326	341			

The majority of the positive results were from unvaccinated respondents flowed by those with complete vaccination but yet to receive booster doses

^*^Statistically significant *p* < 0.05

Remarkably, from Table 9, 5 out of the 15 respondents that tested positive for COVID-19, had been fully vaccinated. However, all 5 of them did not practice safety measures after vaccination. None of the respondents who practised safety measures after vaccination tested positive for covid-19. The remaining 10 respondents that tested positive for COVID-19 had not been vaccinated though they practised safety precautions. This showed the importance of practising post-vaccination safety measures.

**Table 9:** Descriptive crosstab of vaccination status, COVID-19 post-vaccination test result in the practice of safety measures after vaccination

Have you taken covid-19 vaccine ^*^ did you practice safety measures after vaccination? ^*^ post-vaccination COVID-19 **test result crosstabulation**					
Count					
Post-vaccination COVID-19 test result			Did you practice safety measures after vaccination?		Total
			Yes	No	
Positive	Have you taken COVID-19 vaccine	Yes	0	5	5
		No	10	0	10
	Total		10	5	15
Negative	Have you taken COVID-19 vaccine	Yes	141	75	216
		No	110	0	110
	Total		251	75	326
Total	Have you taken COVID-19 vaccine	Yes	141	80	221
		No	120	0	120
	Total		261	80	341

All the unvaccinated respondents reported had continued the practice of safety measures while 71.5% of fully vaccinated respondents and 30.9% of respondents that had received a booster dose practiced post-vaccinated safety measures. This shows a significant relationship between the number of doses received and the practice of COVID-19 safety measures (χ^2^ = 87.914, df = 2, *p*-value = 0.000)

According to the WHO, before the introduction of COVID-19 vaccines, HCWs accounted for 14% of COVID-19 cases [28]. Though, the risk of BTIs among HCWs is said to have declined after the introduction of COVID-19 vaccinations, with a majority of the infected population due to community exposure. BTIs still pose a risk to Patients and HCWs. Hence, the need for screening and testing in this population and above all, the continued practice of safety precautions even after vaccination.

## Limitations

The study used a rapid diagnostic kit for the detection of COVID-19 infection. Though this kit had a diagnostic accuracy of 94.59% [29].

## Conclusion

The study revealed a statistically significant relationship between vaccination status and post-vaccination test results, with a lower incidence of COVID-19 infection among vaccinated HCWs compared to the unvaccinated. Vaccine combinations did not show a significant association with test results, but breakthrough infections were noted, particularly with the Johnson and Johnson vaccine.

Additionally, the timing of vaccination completion demonstrated a significant relationship with post-vaccination testing, highlighting a higher incidence of positive cases within one month of the last vaccine dose. The study also underscored the importance of booster doses, with fully vaccinated individuals showing a lower incidence of breakthrough infections when boosters were administered.

The vaccine breakthrough infection rate in this study was 2%. Considering that 8% of unvaccinated respondents tested positive for COVID-19, showed that the vaccine confers a reasonable level of protection on vaccinated individuals.

A combination of complete vaccination with the necessary booster dose and the practice of safety precautions like regular hand Washing and the use of hand sanitisers were quite effective and efficient in preventing the COVID-19 9 infection.

As the World eases on the Public Health importance of COVID-19 and the necessity to get vaccinated, it is time for us to sustain the ethics of universal precautions (the practice of safety precautions) and encourage continued vaccination exercise as this can help prevent other strains of the SARS COVID-19 virus.

The study contributes valuable insights to the evolving landscape of COVID-19 vaccination, breakthrough infections, and the role of safety measures in preventing transmission among healthcare professionals. These findings underscore the importance of sustained efforts in mitigating the impact of COVID-19 and ensuring the safety of both Healthcare Workers and the broader community.

## Recommendation

Now more than ever as we embrace the new normal and live with COVID-19, every eligible non-vaccinated individual should seek to be vaccinated. Partially vaccinated persons should seek to be fully vaccinated while fully vaccinated individuals should receive the necessary booster dose(s). Overall, everyone should sustain the practice of safety precautions alongside vaccination. To stop the spread of COVID-19, vaccination along with other COVID-19 appropriate behaviours (safety precautions), will keep ourselves and others around us safe.

### Supplementary Information

**Supplementary Material 1.**

## Data Availability

All the data and material used in this research are available upon request from the corresponding Author.
