# Supplementary material for: SARS-CoV-2 vaccine breakthrough infection and the evaluation of safety precaution practice before and after vaccination among healthcare workers in South West, Nigeria
Source: BMC Public Health. 2024 May 8;24:1259. doi: 10.1186/s12889-024-18663-y (PMC11077696; doi:10.1186/s12889-024-18663-y)
Supplement: Supplementary file 1 — Supplementary Material 1. [file 12889_2024_18663_MOESM1_ESM.docx]

**SARS-CoV-2 vaccine breakthrough infection and the evaluation of safety precaution practice before and after vaccination among healthcare workers in South West, Nigeria.**

I am Oluwatosin Idowu Oni, from the Ondo State Primary Health Care Development Agency, Oke-Eda, Akure. This questionnaire was designed to assess the incidence of COVID-19 infection among vaccinated Health Workers and the possible changes in the attitude of these health workers to COVID-19 safety precautions before and after vaccination.

It is the intention of the Corresponding Researcher, Oluwatosin Oni and other Researchers involved to submit the paper for publication.

You do not have to answer any question you are not comfortable with and you can end the interview at any time. Sincere and objective responses are required as any information you give will be confidential. If you have any question about the survey, do not hesitate to ask.

**CONSENT:** Kindly sign below to indicate that you willingly chose to participate in this survey without any form of coercion.

Signature:………………………. Date:………………………..

**Section 1: Socio Demographic Characteristics & Background Information**

| S/N | QUESTION NO | RESPONSE | CODE NO |  |
| --- | --- | --- | --- | --- |
| 1 | Age of the respondent | <20  21-25  26-30  31-35  36-40  >40years | 1  2  3  4  5  6 |  |
| 2 | Marital Status | Single  Married  Divorced  Widowed  Separated | 1  2  3  4  5 |  |
| 3. | Gender | Male  Female  Others (Specify) | 1  2  99 |  |
| 6 | Years of experience | 1-3 years  4-6 years  7-9 years  >10 years | 1  2  3  4 |  |
| 7 | Cadre of respondent | Doctor  Pharmacist  Nurse/Midwife  Lab Scientist/technician  CHO  CHEW | 1  2  3  4  5  6 |  |

**Section 2: Knowledge and practice safety precautions before and after covid-19 vaccination**

| **S/N** | **QUESTION** | **RESPONSE** | **CODE NO** |  |
| --- | --- | --- | --- | --- |
| 8 | Have you ever heard about COVID-19? | Yes  No | 1  2 |  |
| 9 | What are the preventive measures against COVID-19 | Physical distance  Use of nose mask  Use of hand sanitizer/regular washing of hand  Vaccination  Others: ………………. | 1  2  3  4  5 |  |
| 10. | Have you heard about covid-19 vaccine before? | Yes  No  Don’t know | 1  2  3 |  |
| 11. | What is your perception of Covid-19 vaccine | Highly potent  Moderately potent  Not potent  I don’t know | 1  2  3  4 |  |
| 12. | Have you received the covid-19 vaccine? | Yes  No  Don’t know | 1  2  3 |  |
| 13. | How many doses did you receive? | 0 dose (not vaccinated)  1 dose (partially vaccinated)  2 doses (fully vaccinated)  Booster dose | 1  2  3  4 |  |
| 14 | Did you practice safety precautions before vaccination? | Yes  No  Don’t know | 1  2  3 |  |
| 15 | Which safety precautions did you practice | Physical distance  Use of nose mask  Use of hand sanitizer/regular washing of hand  Vaccination  Others: ………………. | 1  2  3  4  5 |  |
| 16 | When did you receive the last dose | < 1 month  1-3 months  4-6 months  >6 months | 1  2  3  4 |  |
| 17 | Which vaccine did you receive | Astrazeneca + Astrazeneca  Astrazeneca + Pfizer  Moderna + Moderna  Pfizer + Pfizer  Johnson + Johnson (1 dose)  Astrazeneca + Astrazeneca +Pfizer  Moderna + moderna +Pfizer  Johnson and johnson + Pfizer  Astrazeneca + Pfizer + Pfizer | 1  2  3  4  5  6  7  8  9  10 |  |
| 18 | Did you practice safety measures after vaccination? | Yes  No  Don’t know | 1  2  3 |  |
| 19 | If “Yes” to above, which safety measures did you practice | Physical distance  Use of nose mask  Use of hand sanitizer/regular washing of hand  Vaccination  Others: ………………. | 1  2  3  4  5 |  |
| 20. | Do you agree to partake of covid-19 test? | Yes  No | 1  2 |  |
| 21. | Was Nasopharyngeal sample collected? | Yes  No | 1  2 |  |
| 22. | Test result of covid-19 | Positive  Negative | 1  2 |  |
